# Access to an optimal treatment. Current situation

**DOI:** 10.1007/s10067-015-3018-7

**Published:** 2015-07-19

**Authors:** Manuel F. Ugarte-Gil, Adriana M. R. Silvestre, Bernardo A. Pons-Estel

**Affiliations:** Servicio de Reumatología, Hospital Nacional Guillermo Almenara Irigoyen, EsSalud, Lima, Peru; Universidad Científica del Sur, Lima, Peru; Ministerio de Salud de la Provincia de Santa Fe, Rosario, Argentina; Servicio de Reumatología, Instituto Cardiovascular de Rosario, Rosario, Argentina

**Keywords:** Rheumatoid arthritis, Drug therapy, Health service accessibility, Access to healthcare

## Abstract

Access to an optimal treatment is determined by several factors, like availability, pricing/funding, and acceptability. In Latin America (LA), one of the regions with more disparities particularly on healthcare in the world, access is affected by other factors, including socio-demographic factors like poverty, living in rural regions, and/or health coverage. Regarding rheumatoid arthritis (RA), an inadequate access to specialists leads to diagnosis and treatment delays diminishing the probability of remission or control. Unfortunately, in almost every LA country, there are cities with more than 100,000 inhabitants without rheumatologists; furthermore, a primary care reference system is present in only about half the countries. In the public health system, coverage of biologic disease-modifying antirheumatic drugs occurs for less than 10 % of the patients in about half of the countries. Also, as healthcare providers based their funding decisions mainly in direct costs instead of on patient-centered healthcare quality indicators, access to new drugs is more complicated in this region than in high-income countries. More accurate epidemiological data from LA need to be obtained in order to improve the management of patients with rheumatic diseases in general and RA in particular.

## Introduction

The World Health Organization has defined access and use of healthcare services as one of the determinants of health [1]; in rheumatoid arthritis (RA), patient-centered healthcare quality indicators (HCQI) include access to care and pharmacological treatment [2].

RA is a chronic autoimmune disease, which may lead to functional disability resulting in high personal and societal economic impact. Access to optimal treatments is needed in order to reduce this burden. Early diagnosis and treatment are necessary to achieve optimal outcomes; early treatment has been associated with a 33 % reduction of radiographic progression compared to delayed treatment [3]. Furthermore, combination therapy and intensive treatment like tight control or treat-to-target (T2T) have been associated with a lower rate of disease progression than monotherapy or non-T2T approaches [4, 5]. These data indicate that RA patients need an efficient healthcare system, which should include an early primary care referral base, well-equipped laboratories and imaging departments, access to a health professional team led by a rheumatologist, and several treatment options [including synthetic and biologic disease-modifying antirheumatic drugs (DMARDs)]. In general, the worldwide Joint Learning Initiative has proposed that overall 2.5 healthcare workers per 1000 inhabitants is an acceptable standard [6]. Regarding rheumatic diseases, at least in the UK, the recommended relationship between rheumatologists and inhabitants is 1/85,000 [7].

In this review, we will examine the importance of an adequate access to treatment and the current situation in Latin America (LA).

## Determinants of access

**Fig. 1:**
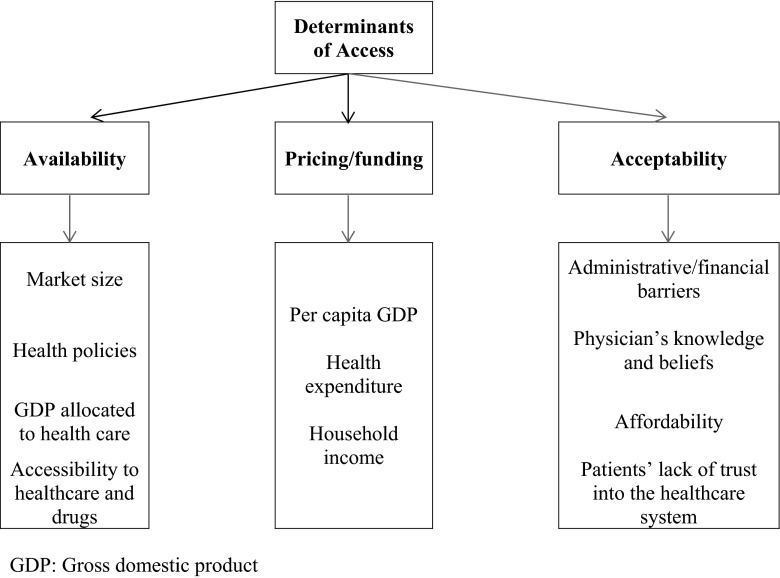
Factors associated with determinants of access

Access to treatment is determined by several factors, including availability, pricing/funding, and acceptability (Fig. 1). Availability is affected by market size and health policies related to low income and a low percentage of the gross domestic product (GDP) allocated to health budgets. In a recent report from Europe, non-members of the European Union (EU) had a lower number of biologic DMARDs approved and even a lower number of biologic DMARDs reimbursed [8]. In addition, the numbers of biologic DMARDs approved and reimbursed are positively correlated with the per capita GDP, median income, and total health expenditures [8]. If we extrapolate these data to LA, it should be expected that overall they will have a lower number of biologic DMARDs approved and reimbursed. Availability also relates to accessibility to healthcare and to drugs; in Portugal, key barriers for access to healthcare and treatment (particularly biologic DMARDs) were found to be related to accessibility to primary care services, difficulties in the diagnosis of RA by general practitioners, an inefficient referral system, and a cumbersome administrative process for the prescription of biologic DMARDs [9]. In Greece, 26 % of the patients had obstacles in accessing a rheumatologist, mainly due to delays in scheduling an appointment or difficulties in traveling to and from the clinic. On the other hand, 49 % of the patients had problems in accessing their medications, mainly due to administrative difficulties and drug non-availability [10]. In Canada, of patients referred with probably RA, possible RA or probably osteoarthritis, about one third were evaluated within 3 months of the referral, another third beyond the first 3 months and the last third were refused evaluation because rheumatologists were not accepting new patients at the time of the referral. Overall, 40 % of patients with possible or probable RA were evaluated by a rheumatologist within 3 months of the referral from the primary care provider. This percentage decreased to 30 % for patients living in small towns [11].

Pricing/funding is particularly important in low-income countries; for example, in eastern European countries, the ratio between the cost of biologic DMARDs and health expenditures per capita was between three- and six-fold higher than in western European countries [12]. Furthermore, the average price, adjusted for the countries’ purchasing power parity, as well as the average expenditure per patient were negatively associated with the per capita GDP, median income, and total health expenditure. Also, the average price and average per patient expenditure positively correlated with disease activity and disability, as measured by the disease activity score (DAS28) and the health assessment questionnaire (HAQ), respectively [8]. In Europe, also, the proportion of patients using biologic DMARDs correlated with the GDP, and this proportion ranged from 7 % (Portugal) to more than 30 % in Ireland and the Netherlands; Portugal had a GDP of USD 23,100 and Ireland and the Netherlands of around USD 40,000 [13]. In a comparison between RA databases, the initiation of anti-TNF compounds seems to be related to reimbursement and physician practices/preferences [14]. A higher household income is associated with a higher probability of starting a biologic DMARD [15]. Even more disease activity is negatively associated with the GDP per capita [16, 17]. These data, taken together, suggest that living in a low-income country should be considered a risk factor for bad prognosis for RA patients.

Acceptability is a more complex concept, and it includes physician’ and patient’s barriers. In the case of physician’s barriers, they could be affected by the healthcare system, including administrative and financial constraints and by the physician’s knowledge and beliefs about biologic DMARDs. For example, even though current guidelines recommend low disease activity as a goal to achieve for all RA patients, more than one third of the patients with moderate or high disease activity did not have a modification on their DMARD therapy in Australia, being the main reasons for not making any modification the presence of irreversible joint damage (19.7 %), patient-driven under-treatment (14.7 %), and rheumatologist-driven under-treatment (9.9 %) [18]. Patient’s barriers include access to the healthcare system (in particular to the rheumatologist), affordability (amount of individual contribution to treatment), and lack of trust in the system and on the drugs (including some region-related myths). In Europe, administrative/financial barriers for prescribers and patients’ lack of trust into the healthcare system are negatively associated with the per capita GDP, median income, and total health expenditure, but other factors of acceptability are not [8].

## Demographic factor related to access to treatment

Access to healthcare is also affected by patients’ demographic factors; for example, patients older than 65 years have less knowledge about the nature and prognosis of the disease and the side effects of the medications [19]. Regarding healthcare utilization, medical specialist services are more frequently used by younger patients, those with longer disease duration, with poorer functional status, and with a high number of comorbidities [20]. Furthermore, cost-related non-adherence in older patients is associated with lower income and lower prescription coverage and is higher in RA patients than in patients with other chronic conditions. In addition, RA patients invest less in basic needs in order to afford their medications [21].

Lower level of education has been associated with higher disease activity in a multinational study [17]. Patients with a low level of education also have less knowledge about the nature and prognosis of the disease and side effects of the drugs [19] and use biologic DMARDs less frequently than those with higher levels of education [22]. In LA, low level of education has been associated with less treatment adherence including patient difficulties in remembering the doses of their medications and/or how frequently they should take them, forgetting to take their medications, and/or decreasing the dosages prescribed [23]. Furthermore, low level of health literacy has been associated with higher level of disease activity in multicenter study in Argentina [24]. The largest early RA multiethnic cohort from LA (*Grupo Latino Americano de Estudio de Artritis Reumatoide*, GLADAR) has shown that almost 60 % of the patients are from the low and middle-low socioeconomic levels and that lower socioeconomic status is associated with higher baseline disease activity [25], but with less use of biologic DMARDs [22].

## Strategies related to access to treatment

### Primary care for RA patients

To achieve the goal of early diagnosis and treatment for all RA patients, an efficient referral system is needed. RA patient who receive care by an internist or a primary care physician is less likely to receive a DMARD than those who see a rheumatologist. In addition, those patients treated by rheumatologists, in particular those treated continuously, use more consistently DMARDs and are more frequently on a combination therapy. Even more so, methotrexate is more commonly used among those patients treated continuously by a rheumatologist [26]. Only 60 % of primary care physicians considered that DMARDs should be started within the first 6 months of diagnosis, 61 % felt uncomfortable starting DMARDs, and 71 % were likely to refer RA patients to specialists, although the most frequent reason for the referral was advanced disease (80 %). Among the reasons for not referring, 40 % did not refer due to insurance problems, 27 % considered a rheumatology appointment too difficult to obtain, and 24 % considered patient did not need a referral [27]. This lack of accessibility to a rheumatologist and the inadequate treatment by internists and/or primary care physicians carries a poor prognosis for these patients. In Canada, access to a rheumatologist for patients with inflammatory arthritis and any arthritis was found to be associated with a better socioeconomic status and access to primary care physician [28].

In order to improve access to rheumatologists, several strategies have been developed. One of them is to use the American College of Rheumatology/European League against Rheumatism (ACR/EULAR) criteria as a prioritization tool. Using it, the time for referral could be reduced to 8 weeks compared to 45 weeks for those patients who did not meet these criteria which gave a sensitivity of 96 %, a specificity of 56 %, a positive predictive value of 40 %, and a negative predictive value of 98 % [29]. Another strategy is the use of a questionnaire administered by a nurse practitioner; based on it, the decision of which patients referred by general practitioner requires an urgent referral; this strategy had a sensitivity of 97 %, a specificity of 55 %, a positive predictive value of 49 %, and a negative predictive value of 97 % [30]. A self-administered tool, which could lead to earlier references without increasing the number of primary care health professionals, had a higher specificity (87 %) but a lower sensitivity of 86 % [31].

### Patient-centered healthcare quality indicators

Access to treatment should be defined not only by the physician’s opinion but should also include the patient’s preferences and needs. In order to do that, patient-centered healthcare quality indicators (HCQIs) have recently been proposed for the provision of healthcare to RA patients. A final set of 14 indicators has been developed to be used in quality improvement and benchmarking in countries across Europe. Among them, two indicators were established for structure (patient information and calculation of composite scores), 11 for process (e.g., access to care, assessments, and pharmacological and non-pharmacological treatments), and one for outcome (effect of treatment on disease activity, including the goal of at least low disease activity after 6 months of treatment). Regarding process indicators, they include the time between the onset of symptoms and the evaluation by specialist (it should be not more than 6 weeks), the time between the onset of symptoms and a tailored education by health professionals as well as an individualized exercise program (not more than 3 months), the time between orthopedic surgeon evaluation, and the presence of joint damage/soft tissue problems that may require surgery (not more than 3 months); others in this category are assessment of disease activity, damage, quality of life, labor force participation, response to treatment (and adjustment according to EULAR guidelines) [2]. Unfortunately, in LA, these indicators are barely accomplished due to the well-documented delays between the primary-care and the specialist’s evaluation.

### Treat to target, tight control strategies

Another pathway to use more efficiently scarce resources is to define treatment’s goals and strategies and follow guidelines which suggest how the treatment should be increased or decreased. Treatment strategies have been changed in the last few years due to the existence of new drug- and protocol-driven studies. These protocol-driven studies include an intensive treatment adjusted according to a predefined outcome measured by a composite disease activity index (usually remission or low disease activity). The systematic use of a disease activity index has led to a high proportion of patients achieving low levels of disease activity compared to those patients in whom this index had not been used [32]. Among protocol-driven studies, the tight control for rheumatoid arthritis (TICORA) study compared routine care against intensive care (monthly assessments and if low disease activity was not achieved, treatment was increased). Patients from the intensive care group had a higher rate of remission and less radiographic progression than those in the routine care group [33]. The Computer-Assisted Management in Early Rheumatoid Arthritis (CAMERA) study evaluated a computerized decision-making program of monthly evaluations for the intensive care group with a predefined treatment scheme and every 3 months for the conventional treatment group; treatment adjustments were done according to joint counts and assessors’ judgment in this group. After 2 years, the intensive care group had a higher percentage of remission, but a similar radiographic progression than the routine care group [34]. In Colombia, the T2T strategy has shown a decrease in disease activity in 80 % of the patients using traditional DMARDs [35], and in Brazil, the T2T strategy was associated with 20.4 % of remission, compared to 12.6 % before using T2T strategy [36].

According to these studies, tight control proved to be associated with a better treatment response and it should be included as a predefined end-point by rheumatologists until it is achieved. According to EULAR recommendations “treatment should be aimed at reaching a target of remission or low disease activity as soon as possible in every patient; and as long as the target has not been reached, treatment should be adjusted by frequent (every 1–3 months) and strict monitoring” [37, 38].

Protocol-driven studies have also lead to the proposal of the T2T strategy which included an end-point (remission or low disease activity) and more frequent evaluations (monthly) when the patient has moderate or high disease activity or less frequent when the goal is achieved [39].

### Cost/effectiveness and cost/utility

Cost/effectiveness is a decision-making criterion for the majority of health insurance systems; however, it is important to point out that several outcomes have been chosen in order to define effectiveness. One of the most used is the quality-adjusted life-year (QALY), which combines life expectancy and quality of life (QoL) by weighting life-years with a quality index [40]. However, as QoL is influenced by disease activity and damage, the earlier the intervention is performed, the higher the opportunity of a better QoL. Furthermore, an official threshold to be considered “cost/effective” has not been established, but if 50,000 € per QALY (a commonly accepted threshold) is considered, biologic DMARDs do achieve this goal [40].

Cost analyses are complex, because they should not include merely direct costs. RA-related costs include productivity losses and, even, the cost of the investment through education, work experience, and job training; this approach is named “the human capital method.” Using this method, the cost of biologic DMARDS could be compensated by savings in productivity [41].

The majority of the cost/effectiveness studies have been performed in the developed world. In low-income countries, this type of analysis is more complicated because the cost of the drugs is similar; yet, the return by improving worker participation would be lower (due to the lower labor value). This situation could lead to a vicious circle, which makes even more complicated the access to an adequate treatment for RA patients in these countries. In addition, even in developed countries, cost/effectiveness and cost/utility evidence is not weighted in the same way, and official drugs’ recommendations include other factors that alter an adequate use of high-quality evidence [42].

Currently, almost all health insurance systems use cost/effectiveness as the only criterion in order to define which patients should or should not use biologic DMARDs; however, if indirect costs are not included, this strategy turns into a barrier for an access to an optimal treatment.

### Latin America. Current situation

Several economic- and healthcare system-related factors are associated with access to treatment, and these factors are of particular interest in LA. Gross national income per capita, adjusted by purchasing power parity in LA, is just 25 % of the USA (USD 12,086 vs. 50,610, respectively), but in Haiti, it is only USD 1240; it means that the cost of current biological treatments per year is higher than the average income per capita in LA. The quintile with the higher income level earns 16 times more per year than those of the lower quintile. This difference is even more dramatic in Panama where this relationship is almost 30 [43].

There are high disparities in terms of the healthcare system between and within the different LA countries. For example, the proportion of medical doctors per 10,000 inhabitants for the entire region is 17.5. As an average, this proportion is adequate; however, it ranges from 4.2 for Bolivia to 68.1 for Cuba. Thus, the distribution of physician within LA needs to improve, and, in some countries, their actual number needs to increase. Among LA countries, the proportion of rheumatologists per 100,000 inhabitants was 0.7 (range from 0.1 for Nicaragua to 3.2 for Uruguay); furthermore, only Argentina and Uruguay had more than 1 rheumatologist per 85,000 inhabitants [44, 45]; these data are depicted in Table 1. Likewise, the health system coverage ranges from 22 % for Paraguay to 100 % for Argentina, Brazil, and Cuba [43]. In addition, the expenditures in the public healthcare system defined as percentage of GDP ranges from 2.4 % in Guatemala to 5.4 % in Uruguay [43] with an average of 3.8 % for the entire region.

**Table 1:** Distribution of rheumatologists across LA countries^a^

Country	Population (million)	Rheumatologists	Proportion of rheumatologist/inhabitants (1/100,000)
Argentina	42.0	850	2.0
Bolivia	10.0	30	0.3
Brazil	200.0	1543	0.8
Chile	17.3	146	0.8
Colombia	44.0	136	0.3
Costa Rica	4.6	21	0.5
Dominican Republic	9.5	18	0.2
Ecuador	14.5	52	0.4
El Salvador	6.7	16	0.2
Guatemala	14.1	32	0.2
Honduras	8.2	13	0.2
Mexico	112.0	568	0.5
Nicaragua	5.3	4	0.1
Panama	3.4	14	0.4
Paraguay	6.5	16	0.2
Peru	29.0	172	0.6
Uruguay	3.3	105	3.2
Venezuela	29.0	157	0.5
Total	559.4	3893	0.7

^a^Adapted from Caballero Uribe CV [44] and Al Maini M et al. [45]

In Mexico, only 19 % of RA patients started DMARD within first 3 months from symptoms onset, and 24 % after 1 year [46]; in Venezuela, mean lag time between symptom onset and the beginning of DMARDs was almost 5 years [47]; in two Argentine centers, from 2004 to 2007, median lag time between symptom onset and the beginning of DMARDs was 4 months but with an interquartile rank from 1 to 24 months [48], and in an Argentine multicenter early arthritis cohort (which began in 2008), all patients with RA started DMARDs within the first 6 months [49]. Almost 50 % of a Mexican early RA cohort did not have adequate treatment compliance, and main reasons were drug availability and costs [50]. Health expenses higher than 30 % of the total house income are associated with a lower level of health coverage and disease duration, and impoverishment is associated with a higher health expense and disability and lower socioeconomic status [51].

Recently, and aiming at this publication, the Pan American League of Associations for Rheumatology (PANLAR) has conducted a survey of LA rheumatologists thru the national societies of the countries from the region; each national society sent the questionnaire to its members. Two hundred and twelve individual responses were obtained and analyzed; this survey reveals that rheumatologists are distributed mainly in urban regions and in large cities; more than 95 % considered that the majority of rheumatologists are in cities with at least 100,000 inhabitants, and a similar percentage considered that there are regions within their countries without access to a specialist. In addition, more than 90 % of participants agreed that there are second and third level hospitals that do not have a rheumatologist and that only 50 % of patients have access to a free evaluation in the public healthcare system. Even more, a primary care reference system only exists in 50 % of the countries, according to the same survey.

Because of the rheumatologists’ geographical distribution in towns and cities, the long distances between them, and the inadequate number of rheumatologists in several LA countries, it would be difficult for RA patients from rural regions or small towns to be evaluated by a rheumatologist; for example, in Costa Rica, there is a 4- to 6-month delay for an evaluation by a rheumatologist which results in patients missing the “window of opportunity” in the treatment of this condition [52].

The availability of traditional DMARDs is almost 100 %, but for the biologic DMARDs including tofacitinib, it ranges from 72.4 % (tofacitinib) to 96.1 % (anti-TNF). Among the 212 LA rheumatologists, almost 50 % consider that the coverage for biologic DMARDs occurs for less than 10 % of the patients in the public healthcare system and within them even worse for tofacitinib. In the social security system, less than 50 % of rheumatologists consider that coverage is over 90 % and among the private insurance system less than 40 % reported coverage over 90 %. Furthermore, less than 60 % of the rheumatologists use T2T or tight control strategies.

Taking all these data together, the access to an optimal treatment for patients with RA in LA is far from being acceptable, situation which is definitively worse for patients of low socioeconomic status.

## Conclusions

1. RA is a chronic inflammatory disease, which leads to disability, having a high individual and societal economic impact. This is particularly true in less-developed countries like those in LA, and in particular in those populations with limited access to healthcare; however, there is scarce information about access to treatment in LA.
2. Inequities in the access to an optimal treatment in LA include not only the lack of drugs, particularly of biological DMARDs, but also less access to specialists which is particularly the case for marginal and rural populations.
3. Early diagnosis and management need to be improved, using prioritization tools for early reference and T2T and tight control strategies until remission or low disease activity are achieved.
4. Determinants of access to treatment, including approval process, pricing decision and funding need to be improved in LA.
5. Health policy about access to treatment should not be based merely on costs but should include patient-centered HCQI as well as the societal impact of disease.
6. In most LA countries, healthcare budgets, by necessity, cover the most frequent pathologies like infectious diseases and maternal/infant problems as priorities leaving chronic diseases including the rheumatic diseases way behind them. In LA, we need more epidemiological and health economic studies to quantify the impact of rheumatic diseases, including RA in the different countries of the region and to achieve a more equitable allocation of funds for the optimal treatment of the population affected.
